# Vortioxetine improves rapid eye movement sleep behavior disorder: A case report

**DOI:** 10.1097/MD.0000000000021003

**Published:** 2020-06-26

**Authors:** Yanli Du, Jiajun Jiang, Chee H. Ng, Lingling Wu, Peifen Zhang, Caixi Xi, Jianbo Lai, Yi Xu, Shaohua Hu, Zheng Wang

**Affiliations:** a Department of Psychiatry, First Affiliated Hospital, Zhejiang University School of Medicine; b Zhejiang University School of Medicine, Hangzhou, China; c Department of Psychiatry, The University of Melbourne, Melbourne, Victoria, Australia; d The Key Laboratory of Mental Disorder's Management of Zhejiang Province, No. 79, Qingchun Road; e Brain Research Institute of Zhejiang University, Hangzhou, China.

**Keywords:** paroxetine, polysomnography, rapid eye movement sleep behavior disorder, vortioxetine

## Abstract

**Rationale:**

Rapid eye movement sleep behavior disorder (RBD) is a kind of sleep disturbance characterized by a loss of normal paralysis of REM sleep with dream enactment behavior during REM sleep. The pharmacotherapy options for treating RBD are limited and the use of antidepressants remains controversial. Further, the role of vortioxetine in RBD has not been evaluated so far.

**Patient concerns:**

A 72-year-old woman presented with recurrent peculiar behaviors such as shouting, punching, kicking or even walking around her bedroom during sleep for over 3 years.

**Diagnosis:**

Clinical examinations and polysomnography indicated the diagnosis of RBD.

**Interventions:**

The patient received treatment with paroxetine and melatonin for 1 year and then paroxetine was discontinued and vortioxetine was initiated in a daily dose of 10 mg.

**Outcomes:**

Treatment with paroxetine and melatonin for one year was ineffective. A trial of vortioxetine 10 mg per day over 3 months resulted in significant clinical improvement.

**Lessons:**

To our knowledge, this is the first reported case of effective treatment of RBD with vortioxetine. Well-designed studies with large samples are needed to verify the clinical benefits.

## 1 Introduction

Rapid eye movement (REM) sleep behavior disorder (RBD) is a parasomnia manifested by a loss of normal muscular atonia and the emergence of complex motor behaviors during REM sleep.^[1]^ In a middle-to-older age population-based sample, the prevalence of RBD was 1.06%.^[2]^ RBD is pathologically characterized by denaturation of alpha-synuclein, which shares the same pathological changes with neurodegenerative diseases such as Parkinson disease (PD), multiple system atrophy and Dementia with Lewy Bodies. It has been reported that RBD patients are at a high risk of developing neurodegenerative diseases, and the conversion rate is approximately 50% in 5 years and 80% in 15 years.^[3,4]^

To date, there is no large-scale randomized controlled trial focusing on the pharmacological intervention of RBD. The American Academy of Sleep Medicine (AASM) drafted a guideline for the evaluation and treatment of RBD based on systematic reviews. Improvement of sleep environment is recommended as level A treatment and drugs including clonazepam and melatonin are recommended as level B.^[5]^ However, the research findings of antidepressant use in treating RBD are contradictory across studies. There was a case report that an RBD patient significantly improved with the use of paroxetine.^[6]^ However, another study suggested that antidepressants, especially selective serotonin reuptake inhibitors, could even induce RBD.^[7]^ More recent studies have reported that the use of antidepressants was strongly associated with REM sleep without atonia, which was attributed to uncovering preclinical symptoms of RBD by antidepressant exposure.^[8,9]^

Vortioxetine, a multi-target antidepressant acting as a regulator of several 5-HT receptor subtypes, can modulate multiple neurotransmitters such as 5-HT, glutamate, gamma-aminobutyric acid, dopamine, norepinephrine, and acetylcholine in brain.^[10]^ Here, we reported a case study of vortioxetine in treating RBD symptoms.

## 2 Case presentation

The 72-year-old Chinese woman attended a major city general hospital because of recurrent peculiar behaviors during sleep for 3 years. At the illness onset, her daughter witnessed the patient shouting, punching, kicking or even walking around her bedroom during sleep about once or twice per month, but the patient had no recollection of her behavior. Her symptoms worsened over time and in the last 12 months, she developed nightmares, dream-enactment behaviors and vocalization during sleep occurring almost nightly. The vivid dreams included violent scenes, and once she dreamt about chasing someone but in fact she was pacing up and down her room. No physical harm towards herself or others was reported.

The laboratory examinations such as routine blood test, liver and kidney function, thyroid hormones, tumor biomarkers, infectious diseases including HIV, syphilis and hepatitis B and C, were all unremarkable. The cranial magnetic resonance imaging revealed scattered ischemic lacunar lesions in bilateral paraventricular and semioval regions. The results of the assessment scales are listed in Table 1. An overnight polysomnography (PSG) showed that the total sleep time: 392.5 minutes; time in bed: 498.0 minutes; sleep efficiency: 78.8%; REM sleep: 73.0 minutes; apnea-hypopnea index: 0. The electromyography monitoring revealed that she had intermittent dynamic chin electromyographic activity and her REM sleep without atonia condition was obvious. Besides, the video also showed that she had trivial limb movements and swing actions during sleep. The patient had no concurrent medicine or alcohol/substance use or any family history of psychiatric diseases or neurodegenerative diseases such as PD. The PSG findings and clinical manifestations met the diagnostic criteria of RBD according to the International Classification of Sleep Disorders (ICSD)-3.^[11]^ She was initially prescribed with paroxetine 20 mg per day and melatonin 3 mg per night. A year later, her daughter noticed that her symptoms had partially improved but she still had nightmares and dream-enactment behaviors such as shouting and crying in sleep for about once a week. As such, paroxetine was discontinued and vortioxetine was initiated in a daily dose of 10 mg.

**Table 1:**
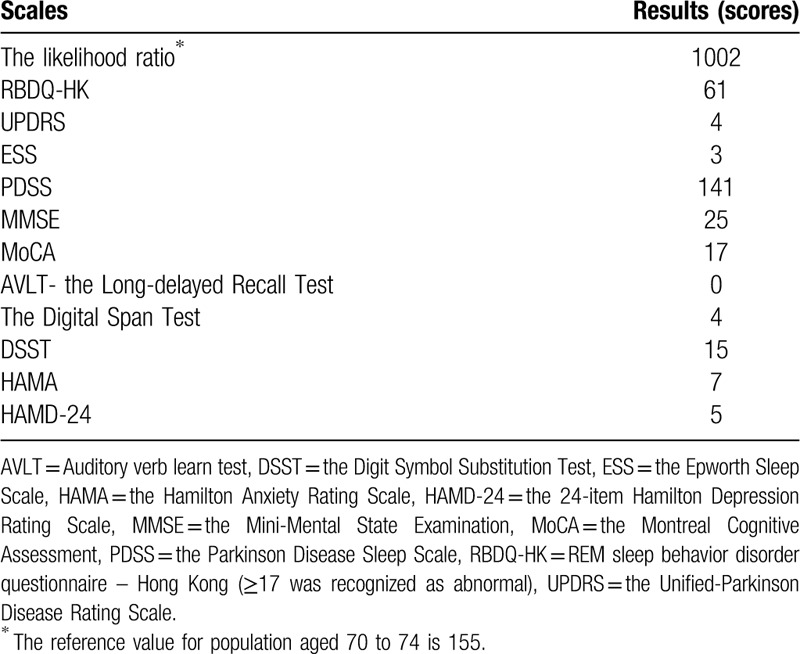
Scores of scales related to risks of rapid eye movement sleep behavior disorder.

In terms of the Clinical Global Impressions Severity scale (CGI-S), score ranges from 1 for “normal” to 7 for “among the most extremely ill” were as follows: at the initial presentation, the CGI-S score was 5; when treated with paroxetine and melatonin for a year, the CGI-S score was rated 4; when treated with vortioxetine for 2 months, the score dropped to 2, and after 3 months of vortioxetine therapy, her CGI-S score dropped to 1. After taking vortioxetine 10 mg per day for 15 months, the CGI-S score of the patient remained 1 point and no obvious side effects of the drug were observed.

This case study was approved by the Institute Ethical Committee of the First Affiliated Hospital, Zhejiang University School of Medicine, and written informed consent was obtained from the patient and her daughter.

## 3 Discussion and conclusions

This is a case report of a female patient with RBD and mild cognitive impairment who responded fully to vortioxetine treatment. She had about 80% chance to develop into prodromal stage of PD through calculating the likelihood ratio.^[12]^ A large-scale multicenter study which assessed the neurodegenerative risk and predictors of neurodegeneration in idiopathic RBD, found that idiopathic RBD had a high risk of developing into PD, Dementia with Lewy Bodies and multiple system atrophy.^[13]^ Therefore, early intervention of RBD is important.

Sara et al. built a preclinical animal model to describe the RBD accurately for the first time and they confirmed that the selective activation of glutamatergic neurons in sublaterodorsal nucleus (SLD) could induce the muscle atonia during REM sleep. While the glutamatergic SLD neuron pathway was genetically inactivated, the rats had abnormal excessive activities and increased muscle tone.^[14]^ The SLD is a region located in the dorsal pons and ventral locus coeruleus, which acts as the trigger of REM sleep. In addition, postmortem studies of brain tissue from RBD patients found the presence of Lewy bodies in/near SLD,^[4]^ indicating that SLD maybe the common target of synucleinopathies and the loss of glutamatergic SLD neurons maybe the primary cause of RBD. As a multimodal antidepressant, vortioxetine could suppress the GABAergic pathway, thus enhancing glutamate neurotransmission in several key brain regions which may alleviate the symptoms of RBD.^[15]^ Besides, studies had indicated that vortioxetine could increase neuroplasticity and dendritic branching more significantly than selective serotonin reuptake inhibitorss such as fluoxetine and escitalopram, which can improve cognitive function.^[10]^

Although one study suggested that 5-HT did not play a role in the progress of RBD directly at brainstem level,^[16]^ many studies has supported that 5-HT is involved in the RBD pathological process. In a preclinical study on PD, Braak et al reported that the pathological change of Lewy body could spread to the raphe nucleus, which is the main resource of serotonin.^[17,18]^ Brain regions such as basal ganglia, hypothalamus, and forebrain that are innervated by serotonergic neurons appear to be involved in the regulation of the sleep-awaken cycle.^[17,18]^ Vortioxetine as a multi-target antidepressant, could regulate the activity of several 5-HT receptor subtypes, including 5-HT_1A_ receptor agonist, 5-HT_3_, 5-HT_7_, and 5-HT_1D_ receptor antagonist, 5-HT_1B_ receptor partial agonist, and inhibition of the 5-HT transporter.^[10]^ Besides, studies have found that the activation of 5-HT_1A_receptor in the brain stem could suppress REM sleep.^[19]^ All this may indicate that vortioxetine may help to relieve the symptoms of RBD, especially through its activation of 5-HT_1A_ receptor.

The recommended pharmacological therapies of RBD are clonazepam and melatonin. However, benzodiazepines can increase the risk of cognitive impairment and withdrawal reactions. RBD is now considered to be precursor marker of neurodegenerative diseases and most RBD patients are of older age. Therefore, clonazepam in RBD needs to be cautiously used. Moreover, although melatonin has a favorable effect on RBD, it is considered as a health product rather than a medication in China, and hence is not well-standardized. In a network meta-analysis that included head-to-head studies of 12 types of antidepressants, vortioxetine showed favorable efficacy and acceptability.^[20]^ A previous study also found that vortioxetine had long-term effects on cognition improvement in patients with major depressive disorder.^[21]^ In an eight-year prospective study, the emergence of RBD symptoms after antidepressants use was considered to be a marker of prodromal neurodegenerative disease rather than side effects of medications. Further, the risk of neurodegeneration even decreased after long-term use of antidepressants.^[22]^

There were several limitations in this case study. Follow up was only for 3 months after using vortioxetine, and long-term follow-up is needed. Besides, the patient did not have another PSG examination after symptom improvement of symptoms.

Considering the mechanism of vortioxetine and clinical outcome of treatment in this RBD patient, future studies to evaluate the role of vortioxetine in treating RBD are justified.

## Author contributions

**Data curation:** Jiajun Jiang.

**Investigation:** Lingling Wu, Peifen Zhang, Caixi Xi.

**Writing – original draft:** Yanli Du.

**Writing – review & editing:** Chee H. Ng, Jianbo Lai, Yi Xu, Shaohua Hu, Zheng Wang.
